# Tuberculosis infection prevention and control in rural Papua New Guinea: an evaluation using the infection prevention and control assessment framework

**DOI:** 10.1186/s13756-023-01237-9

**Published:** 2023-04-12

**Authors:** Gigil Marme, Jerzy Kuzma, Peta-Anne Zimmerman, Neil Harris, Shannon Rutherford

**Affiliations:** 1 Faculty of Medicine and Health Sciences, Department of Public Health and Leadership, Divine Word University, P O Box 483, Madang Province, Papua New Guinea; 2 Faculty of Medicine and Health Sciences, Department of Medicine, Divine Word University, P O Box 483, Madang Province, Papua New Guinea; 3 School of Nursing and Midwifery, Graduate Infection Prevention and Control Program, Griffith University, Parklands Drive, Southport, QLD 4215 Australia; 4 School of Medicine & Dentistry (Public Health), Griffith University, Gold Coast, QLD 4215 Australia

**Keywords:** Tuberculosis, Infection prevention control assessment framework, WHO core components, District hospital, Papua New Guinea

## Abstract

**Background:**

Papua New Guinea (PNG) is one of the 14 countries categorised as having a triple burden of tuberculosis (TB), multidrug-resistant TB (MDR TB), and TB-human immunodeficiency virus (HIV) co-infections. TB infection prevention and control (TB-IPC) guidelines were introduced in 2011 by the National Health Department of PNG. This study assesses the implementation of this policy in a sample of district hospitals in two regions of PNG.

**Methods:**

The implementation of TB-IPC policy was assessed using a survey method based on the World Health Organization (WHO) IPC assessment framework (IPCAF) to implement the WHO’s IPC core components. The study included facility assessment at ten district hospitals and validation observations of TB-IPC practices.

**Results:**

Overall, implementation of IPC and TB-IPC guidelines was inadequate in participating facilities. Though 80% of facilities had an IPC program, many needed more clearly defined IPC objectives, budget allocation, and yearly work plans. In addition, they did not include senior facility managers in the IPC committee. 80% (n = 8 of 10) of hospitals had no IPC training and education; 90% had no IPC committee to support the IPC team; 70% had no surveillance protocols to monitor infections, and only 20% used multimodal strategies for IPC activities. Similarly, 70% of facilities had a TB-IPC program without a proper budget and did not include facility managers in the TB-IPC team; 80% indicated that patient flow poses a risk of TB transmission; 70% had poor ventilation systems; 90% had inadequate isolation rooms; and though 80% have personal protective equipment available, frequent shortages were reported.

**Conclusions:**

The WHO-recommended TB-IPC policy is not effectively implemented in most of the participating district hospitals. Improvements in implementing and disseminating TB-IPC guidelines, monitoring TB-IPC practices, and systematic healthcare worker training are essential to improve TB-IPC guidelines’ operationalisation in health settings to reduce TB prevalence in PNG.

**Supplementary Information:**

The online version contains supplementary material available at 10.1186/s13756-023-01237-9.

## Background

Suboptimal infection prevention and control (IPC) practices in health settings are a major driver of growing antimicrobial resistance and healthcare-associated infections (HAIs). This presents a critical concern for healthcare workers (HCWs) and governments globally [1, 2]. In Europe, one in 18 patients admitted to hospitals and one in 25 in the United States develop HAIs during hospitalisation [3]. In recent decades, the importance of IPC has been overlooked in many health settings worldwide. However, the coronavirus disease (COVID-19) pandemic has highlighted the significance of IPC practices in ensuring the safety of HCWs and patients and reducing the spread into the community [4, 5]. The pandemic has demonstrated that even sophisticated healthcare systems have gaps in implementing IPC measures [6]. Effective IPC strategies such as proper hand hygiene, isolation of patients suspected or confirmed with infectious pathogens, and personal protective equipment (PPE) are essential to minimise HAIs and transmission of infectious disease, thereby underpinning healthcare quality and safety [7].

Effective IPC practices recommended by the World Health Organization (WHO) provide an important framework for IPC programs. These IPC practices are the cornerstones in improving patients’ and HCW’s safety, preventing disease outbreaks, and quality of healthcare in clinical settings worldwide [8]. However, comprehensive information on the assessment of IPC implementation in healthcare settings has been largely limited to high-income settings. In 2018, the WHO developed the IPC Assessment Framework (IPCAF) to strengthen the implementation of their IPC core components in healthcare settings [9]. The IPCAF is a structured closed-formatted survey with an associated scoring system primarily aimed at being self-administered by facility staff. The framework is intended for acute healthcare institutions but can be used in other clinical settings [10].

Recently, the WHO has assessed the implementation of the IPC core components in 81 countries using the IPCAF tool [11]. The WHO assessment found an overall median score of 605 out of 800, demonstrating an advanced level of IPC implementation in many healthcare institutions [11]. However, despite the high IPCAF scores globally, significant variations in the IPC core components remain across different countries [1]. For instance, among the low-income countries studied, only nine (45%) indicated having a national IPC program, four (20%) guidelines on implementation methods, and one (5%) monitored compliance with IPC practice [5]. The global assessment showed that more efforts are needed to strengthen IPC strategies in low-income countries susceptible to disease outbreaks such as COVID-19 and tuberculosis [12].

**Fig. 1:**
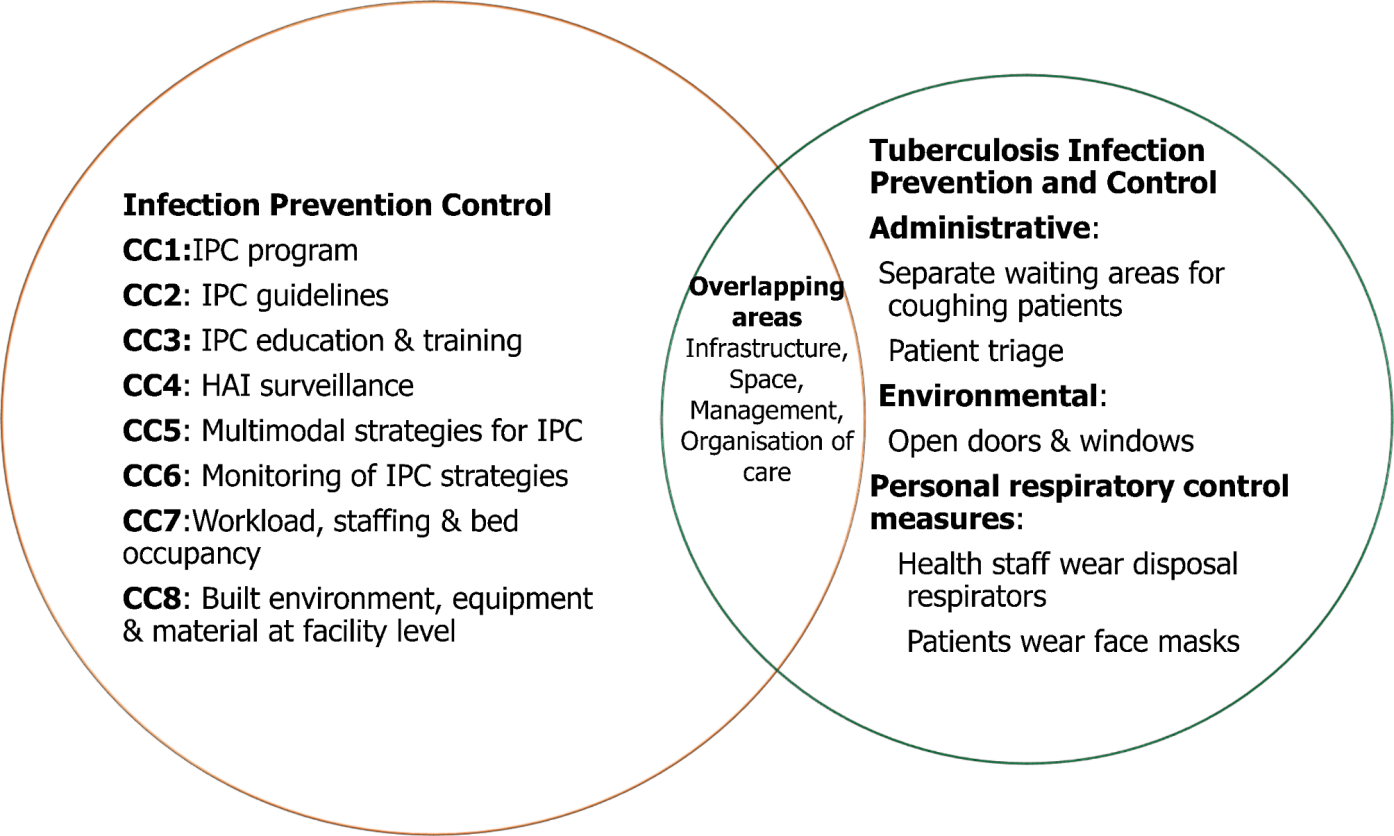
A visual framework of the overlap of broad IPC and TB-IPC guidelines

Tuberculosis infection prevention and control (TB-IPC) is part of the broad IPC strategy. IPC and TB-IPC strategies have overlapping activities such as infrastructure, space, management, and organisation of care. Therefore, establishing an IPC program can influence TB infection control practices and in turn, contribute to the quality of TB care in primary healthcare settings (Fig. 1). The WHO recommends IPC as one of the three measures for reducing the high burden of TB in TB/HIV-prevalent countries [13]. These measures include isoniazid preventive treatment, intensified case finding, and IPC. TB-IPC, such as the administrative, environmental, and respiratory control measures implemented in health settings, can effectively prevent the incidence and prevalence of Mycobacterium TB transmission [14–18]. For example, introducing a cough officer screening (COS) system in a hospital in Taiwan has improved TB detection and prevents TB transmission among healthcare staff, patients and guardians and eventually spread into the community [19].

PNG is among the 14 countries in the world with a triple burden of TB, multidrug-resistant TB (MDR TB), and TB-HIV dual infections [20]. In 2011, TB-IPC was incorporated into the PNG National TB Management plan as policy [21]. Healthcare settings were instructed to implement TB-IPC measures as one of the main strategies to address the TB burden in PNG. However, available data shows that there has been no reduction in TB prevalence despite the requirement to implement this policy in PNG [22, 23]. The number of case notifications of all forms of TB increased between 2008 and 2014 but stabilised during 2015–2016 [22]. In 2019, PNG had an estimated TB incidence of 432 new cases per 100 000 population compared to 333 per 100 000 in 2016 [22, 24]. Since the release of the TB-IPC policy, there has been a limited review of implementation in the PNG health sector. This review is critical to better understand where implementation gaps are to prioritise investment and human resources.

## Methods

### Study design

This survey research was based on a self-administered structured questionnaire adapted from the WHO IPCAF, TB-IPC, and site observations [25]. This design was selected because of its simplicity, affordability, and ease of implementation [26]. This study was approved by the Griffith University Human Research Ethics Committee, Australia, GU Ref No: 2021/921, and PNG Medical Research Advisory Committee (MRAC), MRAC # 22.01. Informed written and verbal consent was obtained from participants during data collection. Approval was also sought from gatekeepers to access health facilities staff and documents.

### Setting and study population

This study was conducted in district hospitals in the PNG Highlands and Momase regions. The district hospitals were targeted because they provide specialised TB services, including sputum examination, radiography, registration of diagnosed cases, and treatment [27, 28]. Of the 23 district hospitals that provide TB services within the two regions, 13 facilities were randomly selected using a random number algorithm available through Excel [29]. The first 13 facilities were included in the study. Out of 13 district hospitals, seven were chosen from the Highlands region and six from the Momase region. However, it was impossible to visit three health facilities for security reasons resulting in a final number of ten facilities, with five from the Highlands and five from the Momase region. The participating health facilities consist of six non-government and four government facilities (Table 1).

### Data collection

#### IPCAF survey instrument

Data collection was conducted from March to June 2022. The implementation of IPC guidelines was evaluated at the health facilities using the structured IPCAF tool [9]. The IPCAF is a validated health facility evaluation tool used globally to assess a facility’s IPC program [11]. The IPCAF evaluates the core components of IPC measured out of 100 points: [1] IPC programs, [2] IPC guidelines, [3] IPC education and training, [4] healthcare-associated infections (HAI) surveillance, [5] multimodal strategies for implementation of IPC interventions, [6] monitoring/audits of IPC practices and feedback, [7] workload, staffing, and bed occupancy and [8] built environment, materials, and equipment for IPC at the facility level, which is inclusive of TB-IPC. This tool uses a four-tier system. Depending on the final score (ranging from 0 to 800), the facility IPC program implementation is grouped into four different IPC categories: inadequate (0-200), basic (201–400), intermediate (401–600), or advanced (601–800).

The IPCAF was also used to assess the implementation of TB-IPC measures including (i) administrative, (ii) environmental, and (iii) personal respiratory measures [9]. This assessment was done through a self-reported survey by facility managers, nursing directors, TB program managers, laboratory workers, and outpatient staff and by observations by the lead researcher of TB-IPC practices in selected locations, including outpatient, laboratory, and TB wards. Based on the overall score achieved in the three TB-IPC measures, the facility was assigned to one of the four TB-IPC levels: inadequate (0–80), basic (80–160), intermediate (160–240), or advanced (240–320).

#### Site observations of TB-IPC practices

An unannounced direct observation of control measures was performed using the WHO TB-IPC health facility assessment checklist to gain an objective assessment of implementation [30]. Observation is considered as an important aspect of data collection to observe the physical and social environment of the research settings including TB ward, isolation room, and laboratory setting. It allows researchers to gain a better knowledge of how activity or program operates as it permits the researcher to witness areas that program employees and participants may omit in an interview [31]. Questions were related to the three TB-IPC measures including administrative, environmental, and personal protective control measures. These observations were conducted between two shifts (morning and evening) [9]. For quality control, the questionnaires and checklists were pilot tested in a non-participating facility and revised accordingly [32].

### Data analysis

Statistical analyses were performed using Statistical Package for the Social Sciences (SPSS) Version 26.0 [33]. Descriptive statistics were conducted for categorical data using univariate frequency analysis and percentages. We summarised the scores by frequency, percentage, and median with the interquartile range [33, 34]. Data from the IPCAF survey were linked with data from facility observations to allow for multidimensional descriptions of TB-IPC components at the facility level. The non-parametric Mann-Whitney U Test was used to compare whether there is a statistical difference or similarity in the median IPC scores between the non-government and government health facilities, which are two independent groups that have implemented TBIPC guidelines [25]. Statistical significance was assessed at p < = 0.05.

## Results

### Health setting information

The assessment included 10 district hospitals covering over 40% (10 of 23) of the Highlands and Momase region facilities. The findings provide a good coverage of non-government and government district hospitals and bed capacity, and the surveyed health facilities are representative of the total sampled district hospitals within the two regions. Among these facilities, the average bed capacity was 60 beds per facility (range of 20 to 120 beds). Six were non-government facilities, and four were government hospitals (Table 1). The profile of the participating health facilities is derived from the Provincial Health Authority (PHA) annual performance reports and health facility websites. A summary of the findings has been provided to the participating district hospitals for quality improvement. The key assessment findings related to health facility-level TB-IPC programs proposed by WHO and the National Department of Health in PNG are discussed in the following sections.

**Table 1:** Profile of participating district hospitals by bed capacity (N = 10)

Region	Type of health facility	Bed capacity	Catchment population
**Momase**	NGO001	80	110,978
**Momase**	NGO002	120	93,107
**Momase**	NGO003	50	81,016
**Highlands**	NGO004	120	101,568
**Highlands**	NGO005	120	126,248
**Highlands**	NGO006	120	39,021
**Momase**	Govt007	48	250,703
**Momase**	Govt008	78	54,672
**Highlands**	Govt009	100	83,036
**Highlands**	Govt010	48	75,067

Key: NGO = non-government organisation, Govt = government

### Distribution of IPCAF score

**Fig. 2:**
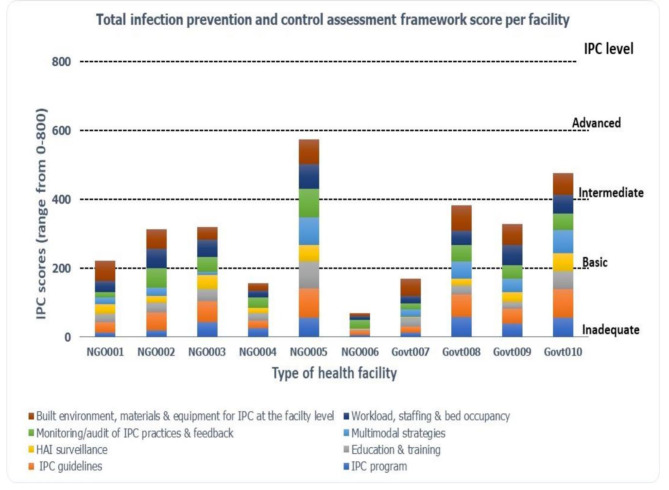
Total IPCAF core component scores by participating district hospitals

The overall median score from the surveyed facilities was 315 out of 800, with an interquartile range of 164.5 to 403.5. The two district hospitals with the highest IPCAF scores (574 and 474) obtained ‘intermediate’ IPC levels according to the WHO IPCAF category. When categorised into IPC level by the score, three (30%) facilities fall into the inadequate (0-200 points) category, five (50%) fall into the basic (201–400) category, and two (20%) facilities fall into the intermediate (401–600) category, with no facilities in the advanced (601–800) category. Overall, the median score for each of the 8 core components varied across each health facility (Fig. 2). The non-parametric Mann-Whitney U Test identified there was no statistically significant difference in the median IPC score between the non-government and government district hospitals (p = 1.00) The data shows that 80% of the health facilities have either inadequate or basic IPC levels. Figure 2 summarises the implementation of IPC core components in the participating health facilities in the Highlands and Momase region, PNG.

### Analysis of infection prevention and control (IPC) implementation in district hospitals

The core components (CC) with the lowest scores and critical areas for attention were: (i) multimodal strategies for implementation of IPC interventions (CC5), (ii) healthcare-associated infections (HAIs) surveillance (CC4), (iii) IPC education and training (CC3), and (vi) IPC program (CC1). Table 2 summarises the distribution of the core components of the surveyed facilities.

**Table 2:** Distribution of IPCAF score by core component

Core component (CC)	Median (IQR)
CC1: IPC program	32.0 (12.0, 55.25)
CC2: IPC guidelines	48.0 (21.75, 70.25)
CC3: IPC education and training	25.0 (19.75, 39.25)
CC4: Healthcare-associated surveillance	24.50 (12.75, 42.50)
CC5: Multimodal strategies for implementation of IPC	22.0 (8.25, 52.75)
CC6: Monitoring/audit of IPC practices and feedback	40.50 (21.50, 51.25)
CC7: Workload, staffing, and bed occupancy	46.50 (21.50, 57.25)
CC8: Environments, materials, and equipment for IPC	57.50 (32.75, 65.50)

#### IPC program (CC1)

The median score for the IPC program was 32.0 (IQR:12.0–55.25). Eight (80%) of the ten district hospitals had an IPC program. However, many IPC programs did not include all the WHO-recommended guidelines, including funding for IPC activities, annual activity plans, staff training, IPC committee, and clearly defined objectives related to local epidemiology. There was also an inadequate representation of senior facility managers in the IPC team and no demonstrable support for IPC objectives and indicators within the facility.

Of the district hospitals included in the survey, 9 (90%) facilities reported no IPC committee comprising a multidisciplinary team actively supporting the IPC team. Further, there were no full-time IPC nurses and inadequate professional development opportunities for IPC practitioners. Only one facility (10%) had access to an adequate microbiology laboratory and provided sufficient quality results on time.

#### IPC education and training (CC3)

CC3 had a median score of 25.0 (IQR: 19.75–39.25). Seven (70%) of the ten facilities do not have expertise in IPC or infectious diseases to lead IPC training. As a result, there is no IPC training for clinical and non-clinical HCWs, including nurses, doctors, administrative and managerial staff, and janitors directly involved with patient care in the facility. Additionally, eight (80%) facilities do not provide ongoing education for IPC staff, including regularly attending conferences and other IPC-related courses. Only two (20%) facilities conducted IPC training for all clinical staff as part of new employee induction and mandatory training.

#### Healthcare-associated infections (HAIs) surveillance (CC4)

CC4 had a median value of 24.50 (IQR: 12.75–42.50). None of the facilities have a person responsible for HAI surveillance activities. Subsequently, HAI surveillance was not conducted in the facilities. Eight (80%) facilities have no established systems for device-associated infections, colonization, or diseases caused by antimicrobial-resistant pathogens. It was also noted that only two (20%) facilities have surveillance protocols, including device-associated infections, local priority epidemic-prone infections (TB and typhoid), and conditions in vulnerable populations. Seven (70%) facilities indicated having no protocol to perform surveillance on HAI and evaluate if the surveillance is in line with the current needs and priorities of the facility.

#### Multimodal strategies for implementation of IPC interventions (CC5)

CC5 received the lowest score in the facility assessment, with a median of 22.0 (IQR: 8.25–52.75). Overall, two (20%) facilities used multimodal strategies such as system change, education and training, monitoring and feedback, communications and reminders, and safety climate and culture change. These two facilities reported ensuring the necessary infrastructure and continuous availability of supplies and addressing ergonomics, and accessibility, such as the best placement of central venous catheter set and tray. Nine (90%) facilities do not have a multidisciplinary team to implement IPC multimodal strategies. They do not regularly link to colleagues from quality improvement and patient safety to promote IPC multimodal strategies.

### Analysis of tuberculosis infection prevention and control (TB-IPC) measures in the facilities

The control measures with the lowest scores and critical areas for attention were environmental and personal respiratory control measures. Table 3 summarises the distribution of the TB-IPC measures in the participating health facilities in the Highlands and Momase region, PNG.

**Table 3:** Distribution of TB-IPC measures

TBIC measures	Median (IQR)
Administrative control measures	235.0 (197.50, 327.50)
Environmental control measures	60.0 (37.25, 85.75)
Personal respiratory control measures	65.0 (42.25, 85.0)

#### Distribution of TB-IPC scores

**Fig. 3:**
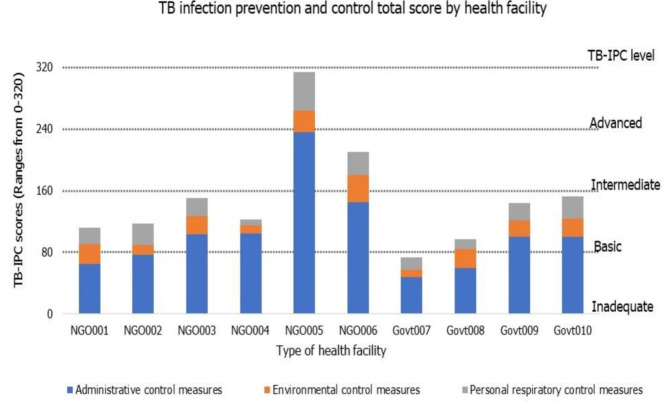
Total TB-IPC scores by participating district hospitals

The overall median score of the TB-IPC was 45.50 out of 320, with an interquartile range between 34 and 53. When categorised by the score into the four TB-IPC implementation levels, one (10%) facility falls into the inadequate (0–80 points) category, seven (70%) fall into the basic ( 80–160) category, one (10%) fall into the intermediate (160–240) category, with one (10%) facility fall in the advanced (240–320) category. Overall, the median score for each of the three core components varied across each health facility. The non-parametric Mann-Whitney U Test identified there was no statistically significant difference in the median TB-IPC score between the non-government and government district hospitals (p = 1.00). The data shows that 80% of the health facilities have either inadequate or basic TBIPC practices. Figure 3 summarises the TB-IPC practices in the participating health facilities in the Highlands and Momase region, PNG.

#### Administrative control measures

The highest median score of 235.0 (IQR: 197.50–327.50) was for the administrative control measures. Of the ten rural hospitals participating in this survey, seven (70%) facilities had a facility TB-IPC plan. However, none of the TB-IPC plans had all the WHO-recommended guidelines, such as a proper budget for TB-IPC activities, TB-IPC committee, staff training on TB-IPC, clearly defined objectives, and work plans based on local epidemiology. Nine (90%) facilities indicated conducting triaging, systematic screening for coughing patients, and regular health education for health workers, patients, and visitors. However, from the site observations, 7 (70%) facilities have not separated coughing patients from other patients. Subsequently, coughing patients congregated with others in the outpatient area, posing a risk of TB transmission to other patients. Additionally, in 8 (80%) facilities, the flow of suspected TB cases through the facility poses a risk for TB transmission. The increasing risk for TB transmission among patients is consistent with the findings from direct observation, where poor physical distancing resulted in overcrowding in the emergency waiting area.

#### Environmental control measures

Environmental control measures had the lowest median score at 60.0 (IQR: 37.27–85.75). None of the facilities surveyed indicated having designated waiting areas for TB patients. Therefore, the available waiting space is for all patients, not TB patients. The inadequate waiting space was consistent with the findings from direct observations where all patients waited in the outpatient waiting areas for medical consultations. However, patients with positive TB who were discharged and returned for review report directly to the TB clinic. The site observations are consistent with this practice in all health facilities.

Eight (80%) facilities indicated using natural ventilation such as opening doors and windows, especially in waiting areas, sputum collection rooms, and patient wards. However, 7 (70%) facilities had poor ventilation systems. The low level of ventilation system in the wards was consistent with the findings from direct observation, where TB ward doors and windows were opened occasionally or not at all. Nine (90%) facilities do not have an isolation room, while five (50%) facilities reported that they do not have a TB ward. Upon site observation in all ten facilities, patients diagnosed with TB are admitted for two months before being discharged with other patients in the medical ward, increasing TB transmission to other potentially immunosuppressed patients.

#### Personal respiratory control measures

Personal respiratory control has the second-highest median score at 65.0 (IQR: 42.25–85.0). Eight (80%) facilities reported having personal protective equipment (PPE) for staff at the health center. However, essential PPE, including N95 respirators and medical face masks, are not continuously available in sufficient quantities for HCWs. The low availability of PPE in health facilities was consistent with the findings from direct observation, where most staff did not use N95 respirators during their consultations with patients. The inadequate PPE has influenced HCWs and patients’ compliance with recommended standards in 7 (70%) facilities. This practice was consistent with the findings from direct observation, where 9 (90%) facilities have inconsistent practices in which HCWs, and patients do not use the recommended PPE during HCW-patient consultations.

## Discussion

This is the first known regional health facility survey using the WHO IPCAF to assess the implementation of IPC and TB-IPC guidelines in PNG district hospitals. The key finding is that most district hospitals had either inadequate or basic IPC and TB-IPC levels. Overall, this study showed that the availability of IPC and TB-IPC programs in the regions does not directly correspond to a well-functioning facility-level TB-IPC practice where main TB-IPC components are implemented.

Specifically, we found challenges related to setting clear objectives and funding for IPC programs. Although many (80%) facilities in the Highlands and Momase region had an IPC program, there was no funding or clear objectives for IPC activities based on the local epidemiology, as the WHO recommends. This result is consistent with the WHO global survey on IPC in healthcare facilities [11] showing that despite the advanced IPC program, overall, only 15.2% of facilities met all indicators considered as minimum criteria for an IPC program [11]. These findings corroborate with other literature evaluating core components of IPC programs. For example, 38 of the 41 (83%) hospitals in Georgia that had an IPC program lacked funding and had unclear objectives [3]. Objectives help define goals, prioritise conflicting activities, guide decision-making, and ensure accountability of resources within the health institution, such as funding. This lack of clear objectives and strategies has led to the neglect of achieving the IPC goals of reducing healthcare-associated infections such as tuberculosis.

Our findings show that specialised IPC and TB-IPC training and education were not offered for HCWs in eight facilities resulting in limited opportunities for ongoing staff development and capacity building. The inadequate capacity building for HCWs in PNG is consistent with a study in Pakistan and Bangladesh that found that limited IPC training among HCWs affected TB-IPC practice. Additionally, TB-IPC training was available for facility managers and senior HCWs but not all staff [13]. Similarly, a study in seven high TB-burden countries found that over half of the HCWs receiving training on TB-IPC guidelines did not understand the content and were unaware of many IPC interventions [36].This point suggests that the quality of training is important. Therefore, standardising training on IPC can achieve more consistent practice nationally supported by adequate educational materials. Mandatory training and resourcing IPC guidelines would strengthen the practice and improve the standard of IPC in the facility. The suboptimal results regarding IPC expertise and inadequate training for HCWs highlight an essential gap and a critical priority area for improvement.

This study shows that few of the Highlands and Momase regions facilities have TB wards, ventilation facilities, and isolation rooms. Limited infrastructure is not unique to PNG; other studies in South Africa, Pakistan, China, and Bangladesh found that inadequate isolation and ventilation systems in healthcare facilities have limited the operationalisation of recommended TB-IPC measures [35, 36]. A similar situation was observed in Ghana, where comparable shortcomings increased TB transmission [37]. A continuing lack of essential health infrastructure, such as a TB ward in district hospitals, will significantly impact TB-IPC strategies and, in turn, the development and transmission of TB among patients, HCWs, and communities. Improving healthcare infrastructure, combined with other methods such as consistent availability of medical equipment and HCWs training, will enhance healthcare quality and prevent TB transmission in healthcare facilities.

Many facilities in this survey reported that PPE was insufficient to adequately protect frontline HCWs. A similar situation was observed in South Africa, where a shortage of PPE in healthcare facilities has seriously affected infection control plans. Additionally, the South African facilities did not provide PPE regularly, and it was supplied to specific departments such as the multi-drug resistant TB (MDR TB) unit [38, 39]. The COVID-19 pandemic has highlighted the significance of IPC in health settings [5]. One important consideration was an investment in PPE to prevent the transmission of COVID-19 among HCWs, patients, and the public. This increased visibility and recognition of PPE should serve to promote the use of such resources and reinforce TB-IPC measures in health settings. This requires regular financial and human investments, including specialised expertise, and highlights the importance of sufficient funding dedicated to IPC.

### Strengths and Limitations

The survey used the WHO IPCAF and allowed for direct comparison with the global study of IPC in healthcare settings. Almost half of the district hospitals in the two regions participated and were randomly selected, allowing careful extrapolations to the regional level. Besides these strengths, the study has several limitations. The IPCAF requires a sound knowledge of the WHO terminology. Participants who self-administered the survey needed to understand unfamiliar terms, including multimodal strategies, creating the potential for misinterpretation and false reporting. This study is limited to the Highlands and Momase regions in PNG. A national survey is therefore recommended to determine the extent of the implementation of TB-IPC across PNG.

## Conclusion

This study assessed the implementation of TB-IPC practice in district hospitals in the Highlands and Momase region, PNG. The study included site assessment followed by validation of practices using the WHO IPCAF survey tool. Our survey findings show a lack of IPC programs, insufficient IPC training and education for HCWs to implement programs, and a lack of IPC infrastructure and personal protective equipment in health facilities. Implementing TB-IPC policy at the provincial level via committees, plans, personnel appointments, improved IPC facilities, and ongoing staff training are effective strategies for improving TB-IPC policy implementation in healthcare institutions.

## Electronic supplementary material

Below is the link to the electronic supplementary material.

Supplementary information 1: Health facility infection prevention and control assessment framework.

Supplementary information 2: TB infection control practice observation checklist.

## Data Availability

The data (health facility assessment survey) are available from the corresponding author upon request.

## Abbreviations

CC: Core component
COVID: Coronavirus disease
GU: Griffith University
HAI: Healthcare associated infections
HCW: Healthcare workers’
IPCAF: Infection Prevention and Control Assessment Framework
MDR TB: Multidrug resistant tuberculosis
PNG MRAC: Papua New Guinea Medical Advisory Research Committee
PNG: Papua New Guinea
TB: Tuberculosis
TB-HIV: Tuberculosis-human immunodeficiency virus
TBIPC: Tuberculosis Infection Prevention and Control
WHO: World Health Organization
