# Supplementary material for: Tuberculosis infection prevention and control in rural Papua New Guinea: an evaluation using the infection prevention and control assessment framework
Source: Antimicrob Resist Infect Control. 2023 Apr 12;12:31. doi: 10.1186/s13756-023-01237-9 (PMC10092912; doi:10.1186/s13756-023-01237-9)
Supplement: Supplementary file 1 — Supplementary information 1: Health facility infection prevention and control assessment framework. [file 13756_2023_1237_MOESM1_ESM.pdf]

Supplementary information 1: Health facility questionnaire

Part A: General assessment of health facility infection prevention and control (IPC) programme

Adopted from 2018 WHO infection prevention and control program assessment framework at a health facility

Goal: To assess the current infection prevention and control (IPC) situation in the health facility , that is, existing IPC activities/resources, and identify strengths and gaps that can inform future plans.

Section 1: Facility demographics

1.1 Name of the health facility	
1.2 Facility type	
1.3 Total catchment population	
1.4 Total TB patient load per year	
1.5 Address	
1.6 District/LLG/Province	
1.7 Region – Momase/Highlands	
1.8 Location: Urban/Rural	
1.9 Name of responsible person for infection control in this facility	
1.10 Participant's name	
1.11 Age	
1.12 Sex	
1.13 Position	
1.14 Contact details	
1.15 Years of work at health facility	
1.16 Educational qualification (MO,HEO,NO,CHW)	
1.17 Facility ownership – Church/Government/Private	

Section 2: Assessment of infection prevention and control programs

Core component 1: Infection Prevention and Control (IPC) programme

Question	Answer	Score	Reasons for score given
1.1 Do you have an IPC programme?[3] Choose one answer	☐ No or don't know	0	
	☐ Yes, without clearly defined objectives	5	
	☐ Yes, with clearly defined objectives <u>and</u> annual activity plan	10	
1.2 Is the IPC programme supported by an IPC team comprising of IPC professionals?[4] Choose one answer	☐ No or don't know	0	
	☐ Not a team, *only* an IPC focal person	5	
	☐ Yes	10	
1.3. Does the IPC team have at least one full-time IPC professional or equivalent	☐ No IPC professional available	0	

(nurse or doctor working 100% in IPC) available?<br>Choose one answer	☐ No, *only* a part-time IPC professional available	2.5	
	☐ Yes, one per > 250 beds	5	
	☐ Yes, one per ≤ 250 beds	10	
1.4. Does the IPC team or focal person have dedicated time for IPC activities?	☐ No	0	
	☐ Yes	10	
1.5. Does the IPC team include both doctors and nurses?	☐ No	0	
	☐ Yes	10	
1.6. Do you have an IPC committee actively supporting the IPC team?	☐ No	0	
	☐ Yes	10	
**1.7. Are any of the following professional groups represented/included in the IPC committee?**			
1.7.1 Senior facility leadership (for example, administrative director, chief executive officer [CEO], medical director)	☐ No or don't know	0	**Reasons for scores given**
	☐ Yes	5<br>0	
1.7.2 Senior clinical staff (for example, physician, nurse)	☐ No or don't know	2.5	
	☐ Yes	0<br>2.5	
1.7.3 Facility management (for example, biosafety, waste, and those tasked with addressing water, sanitation, and hygiene [WASH])	☐ No or don't know	0	
	☐ Yes	2.5	
1.8. Do you have clearly defined IPC objectives in the hospital strategic or annual activity plan (that is, in specific critical areas)?<br>Choose one answer	☐ No or don't know		
	☐ Yes, IPC objectives *only*		
	☐ Yes, IPC objectives and measurable outcome indicators (that is, adequate measures for improvement)	5	
	☐ Yes, IPC objectives, measurable outcome indicators and set future targets	10	
**1.9. Does the senior facility leadership show clear commitment and support for the IPC programme:**			
1.9.1By an allocated budget specifically for the IPC programme (that is, covering IPC activities, including salaries)?	☐ No or don't know	0	
	☐ Yes	5	
1.9.2 By demonstrable support for IPC objectives and indicators within the facility (for example, at executive level meetings, executive rounds, participation in morbidity and mortality meetings)?	☐ No or don't know	0	
	☐ Yes	5	
1.10. Does your facility have microbiological laboratory support (either present on or off site) for routine day-to-day use?<br>Choose one answer	☐ No	0	
	☐ Yes, but not delivering results	5	

	reliably (timely and of sufficient quality)		
	☐ Yes, and delivering results reliably (timely and of sufficient quality)	10	
**Subtotal score**		**/100**	

**Core component 2: Infection Prevention and Control (IPC) guidelines**			
**Question**	**Answer**	**Score**	**Reasons for scores given**
2.1. Does your facility have the expertise (in IPC and/or infectious diseases) for developing or adapting guidelines?	☐ No	0	
	☐ Yes	7.5	
**2.2. Does your facility have guidelines available for:**			
2.2.1 Standard precautions?	☐ No or don't know	0	
	☐ Yes	2.5	
2.2.2 Hand hygiene?	☐ No	0	
	☐ Yes	2.5	
2.2.3 Transmission-based precautions?	☐ No or don't know	0	
	☐ Yes	2.5	
2.2.4 Outbreak management and preparedness?	☐ No or don't know	0	
	☐ Yes	2.5	
2.2.5 Prevention of surgical site infection?[7]	☐ No or don't know	0	
	☐ Yes	2.5	
2.2.6 Prevention of vascular catheter-associated bloodstream infections?	☐ No or don't know	0	
	☐ Yes	2.5	
2.2.7 Prevention of hospital-acquired pneumonia ([HAP]; all types of HAP, including (but not exclusively) ventilator-associated pneumonia)?	☐ No or don't know	0	
	☐ Yes	2.5	
2.2.8 Prevention of catheter-associated urinary tract infections?	☐ No or don't know	0	
	☐ Yes	2.5	
2.2.9 Prevention of transmission of multidrug-resistant (MDR) pathogens?	☐ No or don't know	0	
	☐ Yes	2.5	

2.2.10 Disinfection and sterilization?	☐ No	0	
	☐ Yes	2.5	
2.2.11 Health care worker protection and safety	☐ No	0	
	☐ Yes	2.5	
2.2.12 Injection safety?	☐ No	0	
	☐ Yes	2.5	
.2.2.13 Waste management?	☐ No	0	
	☐ Yes	2.5	
2.2.14 Antibiotic stewardship?	☐ No or don't know	0	
	☐ Yes	2.5	

2.3. Are the infection control guidelines in your facility consistent with national department of health or international guidelines (if they exist)?	☐ No or don't know	0	
	☐ Yes	10	
2.4. Is implementation of the IPC guidelines adapted according to the local needs and resources while maintaining key IPC standards?	☐ No or don't know	0	
	☐ Yes	10	
2.5. Are frontline health care workers involved in <u>both</u> planning and executing the implementation of IPC guidelines in addition to IPC personnel?	☐ No or don't know	0	
	☐ Yes	10	
2.6. Are relevant stakeholders (for example, lead doctors and nurses, hospital managers, quality management) involved in the development and adaptation of the IPC guidelines in addition to IPC personnel?	☐ No or don't know	0	
	☐ Yes	7.5	
2.7. Do health care workers receive specific training related to new or updated IPC guidelines introduced in the facility?	☐ No	0	
	☐ Yes	10	
2.8. Do you regularly monitor the implementation of at least some of the IPC guidelines in your facility?	☐ No or don't know	0	
	☐ Yes	10	
**Subtotal score**		**/100**	

3 IPC team carefully reviews guidelines to prioritize activities according to needs and resources while maintaining key IPC standards.

Core component 3: Infection Prevention and Control (IPC) education and training

Question	Answer	Score	Reasons for scores given
3.1. Are there personnel with the IPC expertise (in IPC and/or infectious diseases) to lead IPC training?	☐ No	0	
	☐ Yes	10	
3.2. Are there additional non-IPC personnel with adequate skills to serve as trainers and mentors (for example, link nurses or doctors, champions)? Choose one answer	☐ No	0	
	☐ Yes	10	
3.3. How frequently do health care workers receive training regarding IPC in your facility? Choose one answer	☐ Never or rarely	0	
	☐ New employee orientation *only* for health care workers	5	
	☐ New employee orientation <u>and</u> regular (at least annually) IPC training for health care workers offered but not mandatory	10	
	☐ New employee orientation <u>and</u> regular (at least annually) mandatory IPC training for all health care workers	15	
3.4. How frequently do cleaners and other personnel directly involved in patient care receive training regarding IPC in your facility? Choose one answer	☐ Never or rarely or don't know	0	
	☐ New employee orientation *only* for other personnel	5	
	☐ New employee orientation <u>and</u> regular (at least annually) training for other personnel offered but not mandatory	10	
	☐ New employee orientation <u>and</u> regular (at least annually) mandatory IPC training for other personnel	15	
3.5. Does administrative and managerial staff receive general training regarding IPC in your facility? Choose one answer	☐ No or don't know	0	
	☐ Yes	5	
3.6. How are health care workers and other personnel trained? Choose one answer	☐ No training available	0	
	☐ Using written information and/or oral instruction and/or e-learning *only*	5	
	☐ Includes *additional* interactive training sessions (for example, simulation and/or bedside training)	10	

3.7. Are there periodic evaluations of the effectiveness of training programmes (for example, hand hygiene audits, other checks on knowledge)? Choose one answer	☐ No or don't know	0	
	☐ Yes, but not regularly	5	
	☐ Yes, regularly (at least annually)	10	
3.8. Is IPC training integrated in the clinical practice and training of other specialties (for example, training of surgeons involves aspects of IPC)? Choose one answer	☐ No or don't know	0	
	☐ Yes, in some disciplines	5	
	☐ Yes, in all disciplines	10	
3.9. Is there specific IPC training for patients or family members to minimize the potential for health care-associated infections (for example, immunosuppressed patients, patients with invasive devices, patients with multidrug-resistant infections)?	☐ No or don't know	0	
	☐ Yes	5	
3.10. Is ongoing development/education offered for IPC staff (for example, by regularly attending conferences, courses)?	☐ No	0	
	☐ Yes	10	
**Subtotal score**		**/100**	

**Core component 4: Health care-associated infection (HAI) surveillance**			
**Question**	**Answer**	**Score**	**Reasons for scores given**
**Organization of surveillance**			
4.1. Is surveillance a defined component of your IPC programme?	☐ No or don't know	0	
	☐ Yes	5	
4.2. Do you have personnel responsible for surveillance activities?	☐ No or don't know	0	
	☐ Yes	5	
4.3. Have the professionals responsible for surveillance activities been trained in basic epidemiology, surveillance and IPC (that is, capacity to oversee surveillance methods, data management and interpretation)?	☐ No or don't know	0	
	☐ Yes	5	
4.4. Do you have informatics/IT support to conduct your surveillance (for example, equipment, mobile technologies, electronic health records)?	☐ No or don't know	0	
	☐ Yes	5	
**Priorities for surveillance - defined according to the scope of care**			
4.5. Do you go through a prioritization exercise to determine the HAIs to be targeted for surveillance according to the local context (that is, identifying infections that are major causes of morbidity and mortality in the facility)?	☐ No or don't know	0	
	☐ Yes	5	
4.6. In your facility is surveillance conducted for:			
4.6.1 Surgical site infections?	☐ No or don't know	0	
	☐ Yes	2.5	
4.6.2 Device-associated infections (for example, catheter-associated urinary tract infections, central line-associated bloodstream infections, peripheral-line associated bloodstream infections, ventilator-associated pneumonia)?	☐ No or don't know	0	
	☐ Yes	2.5	
	☐ No or don't know	0	

4.6.3 Clinically-defined infections (for example, definitions based only on clinical signs or symptoms in the absence of microbiological testing)?	☐ Yes	2.5	
4.6.4 Colonization or infections caused by multidrug-resistant pathogens according to your local epidemiological situation?	☐ No or don't know	0	
	☐ Yes	2.5	
4.6.5 Local priority epidemic-prone infections (for example, norovirus, influenza, tuberculosis [TB], severe acute respiratory syndrome [SARS], Ebola, Lassa fever)?	☐ No or don't know	0	
	☐ Yes	2.5	
4.6.6. Infections in vulnerable populations (for example, neonates, intensive care unit, immunocompromised, burn patients)?[14]	☐ No or don't know	0	
	☐ Yes	2.5	
4.6.7 Infections that may affect health care workers in clinical, laboratory, or other settings (for example, hepatitis B or C, human immunodeficiency virus [HIV], influenza)?	☐ No or don't know	0	
	☐ Yes	2.5	
4. 7. Do you regularly evaluate if your surveillance is in line with the current needs and priorities of your facility?[11]	☐ No or don't know	0	
	☐ Yes	5	

Methods of surveillance

4. 8. Do you use reliable surveillance case definitions (defined numerator and denominator according to international definitions [e.g. CDC NHSN/ECDC] or if adapted, through an evidence-based adaptation process and expert consultation?	☐ No or don't know	0	
	☐ Yes	5	
4. 9. Do you use standardized data collection methods (for example, active prospective surveillance) according to international surveillance protocols (for example, CDC NHSN/ECDC) or if adapted, through an evidence-based adaptation process and expert consultation?	☐ No or don't know	0	
	☐ Yes	5	
4. 10. Do you have processes in place to regularly review data quality (for example, assessment of case report forms, review of microbiology results, denominator determination, etc.)?	☐ No or don't know	0	
	☐ Yes	5	
4. 11. Do you have adequate microbiology and laboratory capacity to support surveillance? Choose one answer	☐ No or don't know	0	
	☐ Yes, can differentiate gram-positive/negative strains <u>but</u> cannot identify pathogens	2.5	
	☐ Yes, can reliably identify pathogens (for example, isolate identification) in a timely manner	5	
	☐ Yes, can reliably identify pathogens <u>and</u> antimicrobial drug resistance	10	

	patterns (that is, susceptibilities) in a timely manner		
**Information analysis and dissemination/data use, linkage, and governance**			
4. 12. Are surveillance data used to make tailored unit/facility-based plans for the improvement of IPC practices?	☐ No or don't know	0	
	☐ Yes	5	
4. 13. Do you analyze antimicrobial drug resistance on a regular basis (for example, quarterly/half-yearly/annually)?	☐ No or don't know	0	
	☐ Yes	5	
4. 14. Do you regularly (for example, quarterly/half-yearly/annually) feedback up-to-date surveillance information to:			
4.14.1 Frontline health care workers (doctors/nurses)?	☐ No or don't know	0	
	☐ Yes	2.5	
4.14.2 Clinical leaders/heads of department	☐ No or don't know	0	
	☐ Yes	2.5	
4.14.3 IPC committee	☐ No or don't know	0	
	☐ Yes	2.5	
4.14.4 Non-clinical management/administration (chief executive officer/chief financial officer)?	☐ No or don't know	0	
	☐ Yes	2.5	
**4**. 15. How do you feedback up-to-date surveillance information?<br>(at least annually)<br>Choose one answer	☐ No or don't know feedback	0	
	☐ By written/oral information *only*	2.5	
	☐ By presentation <u>and</u> interactive problem-orientated solution finding	7.5	
**Subtotal score**		**/100**	

Core component 5: Multimodal strategies for implementation of infection prevention and control (IPC) interventions

Question	Answer	Score	Reasons for scores given
5. 1. Do you use multimodal strategies to implement IPC interventions?	☐ No or don't know	0	
	☐ Yes	15	
5. 2. Do your multimodal strategies include any or all of the following elements: Choose one answer (the most accurate) per element	System change		
	☐ Element not included in multimodal strategies	0	
	☐ Interventions to ensure the necessary infrastructure and continuous availability of supplies are in place	5	
	☐ Interventions to ensure the necessary infrastructure and continuous availability of supplies are in place <u>and</u> addressing ergonomics[17] and accessibility, such as the best placement of central venous catheter set and tray	10	
	**Education and training**		
	☐ Element not included in multimodal strategies	0	
	☐ Written information and/or oral instruction and/or e-learning *only*	5	
	☐ *Additional* interactive training sessions (includes simulation and/or bedside training)	10	
	**Monitoring and feedback**		
	☐ Element not included in multimodal strategies	0	
	☐ Monitoring compliance with process or outcome indicators (for example, audits of hand hygiene or catheter practices)	5	
	☐ Monitoring compliance <u>and</u> providing timely feedback of monitoring results to health care workers and key players	10	
	**Communications and reminders**		
	☐ Element not included in multimodal strategies	0	
	☐ Reminders, posters, or other advocacy/awareness-raising tools to promote the intervention	5	
	☐ *Additional* methods/initiatives to improve team communication across units and disciplines (for example, by establishing regular case conferences and feedback rounds)	10	
	**Safety climate and culture change**		
	☐ Element not included in multimodal strategies	0	
	☐ Managers/leaders show visible support and act as champions and role models, promoting an adaptive	5	

	approach[18] and strengthening a culture that supports IPC, patient safety and quality		
	☐ *Additionally*, teams and individuals are empowered so that they perceive ownership of the intervention (for example, by participatory feedback rounds)	10	

5. 3. Is a multidisciplinary team used to implement IPC multimodal strategies?	☐ No or don't know	0	Reasons for given score
	☐ Yes	15	
5. 4. Do you regularly link to colleagues from quality improvement and patient safety to develop and promote IPC multimodal strategies?	☐ No or don't know	0	
	☐ Yes	10	
5. 5. Do these strategies include bundles or checklists?	☐ No or don't know	0	
	☐ Yes	10	
**Subtotal score**		**/100**	

Core component 6: Monitoring/audit of IPC practices and feedback

Question	Answer	Score	Reasons for given score
6. 1. Do you have trained personnel responsible for monitoring/audit of IPC practices and feedback?	☐ No or don't know	0	
	☐ Yes	10	
6. 2. Do you have a well-defined monitoring plan with clear goals, targets and activities (including tools to collect data in a systematic way)?	☐ No or don't know	0	
	☐ Yes	7.5	
6. 3. Which processes and indicators do you monitor in your facility? Tick all that apply	☐ None	0	
	☐ Hand hygiene compliance (using the WHO hand hygiene observation tool or equivalent)	5	
	☐ Intravascular catheter insertion and/or care	5	
	☐ Wound dressing change	5	
	☐ Transmission-based precautions and isolation to prevent the spread of multidrug resistant organisms (MDRO)	5	
	☐ Cleaning of the ward environment	5	
	☐ Disinfection and sterilization of medical equipment/instruments	5	
	☐ Consumption/usage of alcohol-based handrub or soap	5	
	☐ Consumption/usage of antimicrobial agents	5	
	☐ Waste management	5	
6. 4. How frequently is the *WHO Hand Hygiene Self-Assessment Framework Survey* undertaken? Choose one answer	☐ Never	0	
	☐ Periodically, but no regular schedule	2.5	
	☐ At least annually	5	
6. 5. Do you feedback auditing reports (for example, feedback on hand hygiene compliance data or other processes) on the state of the IPC activities/performance? Tick all that apply	☐ No reporting	0	
	☐ Yes, within the IPC team	2.5	
	☐ Yes, to department leaders and managers in the areas being audited	2.5	
	☐ Yes, to frontline health care workers	2.5	
	☐ Yes, to the IPC committee or quality of care committees or equivalent	2.5	
	☐ Yes, to hospital management and senior administration	2.5	
6.6. Is the reporting of monitoring data undertaken regularly (at least annually)?	☐ No or don't know	0	
	☐ Yes	10	
	☐ No or don't know	0	

6.7. Are monitoring and feedback of IPC processes and indicators performed in a "blame-free" institutional culture aimed at improvement and behavioural change?	☐ Yes	5	
6.8. Do you assess safety cultural factors in your facility (for example, by using other surveys such as HSOPSC, SAQ, PSCHO, HSC)	☐ No or don't know	0	
	☐ Yes	5	
**Subtotal score**		**/100**	

Core component 7: Workload, staffing and bed occupancy

**Question**	**Answer**	**Score**	**Reasons for given score**
**Staffing**			
7.1. Are appropriate staffing levels assessed in your facility according to patient workload using national standards or a standard staffing needs assessment tool such as the *WHO Workload indicators of staffing need*[2] method?	☐ No or don't know	0	
	☐ Yes	5	
7.2. Is an agreed (that is, WHO or national health department) ratio of health care workers to patients maintained across your facility?<br>Choose one answer	☐ No	0	
	☐ Yes, for staff in less than 50% of units	5	
	☐ Yes, for staff in more than 50% of units	10	
	☐ Yes, for all health care workers in the facility	15	
7.3. Is a system in place in your facility to act on the results of the staffing needs assessments when staffing levels are deemed to be too low?	☐ No	0	
	☐ Yes	10	
**Bed occupancy**			
7.4. Is the design of wards in your facility in accordance with international standards regarding bed capacity?<br>Choose one answer	☐ No	0	
	☐ Yes, but *only* in certain departments	5	
	☐ Yes, for all departments (including emergency department and paediatrics)	15	
7.5. Is bed occupancy in your facility kept to one patient per bed?<br>Choose one answer	☐ No	0	
	☐ Yes, but *only* in certain departments	5	
	☐ Yes, for all units (including emergency departments and paediatrics)	15	
7.6. Are patients in your facility placed in beds standing in the corridor outside of the room (including beds in the emergency department)?<br>Choose one answer	☐ Yes, more frequently than twice a week	0	
	☐ Yes, less frequently than twice a week	5	
	☐ No	15	

7.7. Is adequate spacing of > 1 meter between patient beds ensured in your facility?<br>Choose one answer	☐ No	0	
	☐ Yes, <u>but</u> *only* in certain departments	5	
	☐ Yes, for all departments (including emergency department and paediatrics)	15	
7.8. Is a system in place in your facility to assess and respond when adequate bed capacity is exceeded?<br>Choose one answer	☐ No	0	
	☐ Yes, this is the responsibility of the head of department	5	
	☐ Yes, this is the responsibility of the hospital administration/ management	10	
**Subtotal score**		**/100**	

Core component 8: Built environment, materials and equipment for IPC at the facility level

Question	Answer	Score	Reasons for given score
**Water**			
8.1. Are water services available at all times and of sufficient quantity for all uses (for example, hand washing, drinking, personal hygiene, medical activities, sterilization, decontamination, cleaning and laundry)?<br>Choose one answer	☐ No, available on average < 5 days per week	0	
	☐ Yes, available on average ≥ 5 days per week or every day <u>but</u> not of sufficient quantity	2.5	
	☐ Yes, every day <u>and</u> of sufficient quantity	7.5	
8.2. Is a reliable safe drinking water station present and accessible for staff, patients and families at all times and in all locations/wards?<br>Choose one answer	☐ No, not available	0	
	☐ Sometimes, or only in some places or not available for all users	2.5	
	☐ Yes, accessible at all times <u>and</u> for all wards/groups	7.5	
**Hand hygiene and sanitation facilities**			
8.3. Are functioning hand hygiene stations (that is, alcohol-based handrub solution or soap and water and clean single-use towels) available at all points of care?<br>Choose one answer	☐ No, not present	0	
	☐ Yes, stations present, <u>but</u> supplies are not reliably available	2.5<br>7.5<br>0	
	☐ Yes, with reliably available supplies		
8.4. In your facility, are ≥ 4 toilets <u>or</u> improved latrines available for outpatient settings or ≥ 1 per 20 users for inpatient settings?<br>Choose one answer	☐ Less than required number of toilets or latrines available <u>and</u> functioning		
	☐ Sufficient number present <u>but</u> not all functioning	2.5	
	☐ Sufficient number present <u>and</u> functioning	7.5	
**Power supply, ventilation and cleaning**			
8.5. In your health care facility, is sufficient energy/power supply available at day <u>and</u> night for all uses (for example, pumping and boiling water,	☐ No	0	
	☐ Yes, sometimes or only in some of the mentioned areas	2.5	

sterilization and decontamination, incineration or alternative treatment technologies, electronic medical devices, general lighting of areas where health care procedures are performed to ensure safe provision of health care and lighting of toilet facilities and showers)? Choose one answer	☐ Yes, always and in all mentioned areas	5	
8.6. Is functioning environmental ventilation (natural or mechanical) available in patient care areas?	☐ No	0	
	☐ Yes	5	
8.7. For floors and horizontal work surfaces, is there an accessible record of cleaning, signed by the cleaners each day? Choose one answer	☐ No record of floors and surfaces being cleaned	0	
	☐ Record exists, but is not completed and signed daily or is outdated	2.5	
	☐ Yes, record completed and signed daily	5	
8.8. Are appropriate and well-maintained materials for cleaning (for example, detergent, mops, buckets, etc.) available? Choose one answer	☐ No materials available	0	
	☐ Yes, available but not well maintained	2.5	
	☐ Yes, available and well-maintained	5	

Patient placement and personal protective equipment (PPE) in health care settings

8.9. Do you have single patient rooms or rooms for cohorting patients with similar pathogens if the number of isolation rooms is insufficient (for example, TB, measles, cholera, Ebola, SARS)? Choose one answer	☐ No	0	
	☐ No single rooms but rather rooms suitable for patient cohorting available	2.5	
	☐ Yes, single rooms are available	7.5	
8.10. Is PPE available at all times and in sufficient quantity for all uses for all health care workers? Choose one answer	☐ No	0	
	☐ Yes, but not continuously available in sufficient quantities	2.5	
	☐ Yes, continuously available in sufficient quantities	7.5	

Medical waste management and sewage

8.11. Do you have functional waste collection containers for non-infectious (general) waste, infectious waste and, sharps waste in close proximity to all waste generation points? Choose one answer	☐ No bins or separate sharps disposal	0	
	☐ Separate bins present but lids missing or more than 3/4 full; only two bins (instead of three); or bins at some but not all waste generation points	2.5	
	☐ Yes	5	

8.12. Is a functional burial pit/fenced waste dump or municipal pick-up available for disposal of non-infectious (non-hazardous/ general waste)? Choose one answer	☐ No pit or other disposal method used	0	
	☐ Pit in facility but insufficient dimensions; pits/dumps overfilled or not fenced/locked; or irregular municipal waste pick up	2.5	
	☐ Yes	5	
8.13. Is an incinerator or alternative treatment technology for the treatment of infectious and sharp waste (for example, an autoclave) present (either present on or off site and operated by a licensed waste management service), functional and of a sufficient capacity? Choose one answer	☐ No, none present	0	
	☐ Present, but not functional	1	
	☐ Yes	5	
8.14. Is a wastewater treatment system (for example, septic tank followed by drainage pit) present (either on or off site) and functioning reliably? Choose one answer	☐ No, not present	0	
	☐ Yes, but not functioning reliably	2.5	
	☐ Yes and functioning reliably	5	

Decontamination and sterilization

**8.15. Does your health care facility provide a dedicated decontamination area and/or sterile supply department (either present on or off site and operated by a licensed decontamination management service) for the decontamination and sterilization of medical devices and other items/equipment?** Choose one answer	☐ No, not present	0	
	☐ Yes, but not functioning reliably	2.5	
	☐ Yes and functioning reliably	5	
**8.16. Do you reliably have sterile and disinfected equipment ready for use?** Choose one answer	☐ No, available on average < five days per week	0	
	☐ Yes, available on average ≥ five days per week or every day, but not of sufficient quantity	2.5	
	☐ Yes, available every day and of sufficient quantity	5	
**8.17. Are disposable items available when necessary? (for example, injection safety devices, examination gloves)** Choose one answer	☐ No, not available	0	
	☐ Yes, but *only* sometimes available	2.5	
	☐ Yes, continuously available	5	

**Subtotal score**	**/100**

30 Cohorting strategies should be based on a risk assessment conducted by the IPC team.

31 Negative pressure ventilation conditions in isolation rooms may be necessary to prevention transmission of some organisms (for example, multidrug-resistant TB).

32 Personal Protective Equipment (PPE): Medical non-sterile and surgical sterile gloves, surgical masks, goggles or face shields and gowns are considered as essential PPE. Respirators and aprons should also be available in adequate quantities in all facilities for use when necessary.

Interpretation: A three-step process

1. Add up your points

	**Score**
**Section (Core component)**	**Subtotals**
1. IPC programme	
2. IPC guidelines	
3. IPC education and training	
4. HAI surveillance	
5. Multimodal strategies	
6. Monitoring/audits of IPC practices and feedback	
7. Workload, staffing and bed occupancy	
8. Built environment, materials and equipment for IPC at the facility level	
**Final total score**	**/800**

2. Determine the assigned "IPC level" in your facility using the total score from Step 1

**Total score** (range)	**IPC level**
0–200	Inadequate
201–400	Basic
401–600	Intermediate
601–800	Advanced

3. Review the framework results and develop an action plan

Review the areas identified by this evaluation as requiring improvement in your facility and develop an action plan to address them. To undertake this task, consult the WHO *Interim practical manual* supporting implementation of the WHO Guidelines on Core Components of Infection Prevention and Control Programmes[2] which will provide you with guidance, templates, tips, and examples from around the world as well as with a list of relevant IPC improvement tools. Keep a copy of this assessment to compare with repeated uses in the future.

Part B: Assessment of TB infection control measures

Adopted from the WHO 2019 tuberculosis infection prevention and control programme for health facility level

Goal: To assess the current TBIC situation in the health facility, that is, existing TBIC activities/resources, and identify strengths and gaps that can inform future plans.

Section 3: Assessment of tuberculosis infection control measures

**Core component 1: Managerial/Administrative control measures**			
**Question**	**Answer**	**Score**	**Reasons for given score**
1.1. Is there a facility TB infection prevention control management plan in place?[3] Choose one answer	☐ No	0	
	☐ Yes, without clearly defined objectives	5	
	☐ Yes, with clearly defined objectives <u>and</u> annual activity plan	10	
1.2. Is there TB infection control team or responsible person in place? Choose one answer	☐ No	0	
	☐ Not a team, *only* an IPC focal person	5	
	☐ Yes	10	
1.3. Does the TB infection and prevention control team or focal person have dedicated time for TB infection prevention control activities?	☐ No	0	
	☐ Yes	10	
1.4. Is the TB infection control plan part of the facility annual activity plan?	☐ No or don't know	0	
	☐ Yes	10	
1. 5. Is the TBIC plan properly budgeted?	☐ No or don't know	0	
	☐ Yes	10	
~~1.6. Is budget available for TB infection control?~~	~~☐ No or don't know~~	~~0~~	
	~~☐ Yes~~	~~10~~	
1.7. Does TB infection plan include staff training on infection control?	☐ No	0	
	~~☐ Yes~~	~~10~~	
1.8. Is health education on TB infection control ensured for health workers, patients, and visitors?	☐ No	0	
	☐ Yes	10	
~~1.9. Are materials available for infection control?~~	~~☐ No~~	~~0~~	
	~~☐ Yes~~	~~10~~	
	~~☐ Yes~~	~~10~~	
1.11. Is triaging practiced at this rural hospital?	☐ No	0	
	☐ Yes	10	
1.12. Is patient with suspected or positive TB separation practiced at the health facility?	☐ No	0	
	☐ Yes	10	
	☐ No	0	

1.13. Is cough etiquette (cough into elbow, blow into handkerchief, no spiting, social distancing) practiced at the health facility?	☐ Yes	10	
1.14. Is there systematic screening for all patients for cough?	☐ No	0	
	☐ Yes	10	
1.15. Are patients with cough separated early from other patients?	☐ No	0	
	☐ Yes	10	
1.16. Are suspected or diagnosed TB patients separated from suspected or diagnosed HIV patients?	☐ No	0	
	☐ Yes	10	
1.17. Is there a system established to prioritized smear positive cases such as creating an "express lane" to minimise the stay of these patients?	☐ No	0	
	☐ Yes	10	
1.18. Is the flow of TB suspects or patients in the facility a risk for TB transmission?	☐ No	0	
	☐ Yes	10	
1.19. Is there information education and communication (IEC) regarding cough etiquette on site (posters, brochures, signs)?	☐ No	0	
	☐ Yes	10	
1.20. Is the average waiting time/turnaround time for lab investigations such as sputum test within 24 hours?	☐ No or don't know	0	
	☐ Yes	10	
1.21. Are masks, and tissues provided for coughing patients?	☐ No	0	
	☐ Yes	10	
1.22. Are staff checked periodically for TB?	☐ No	0	
	☐ Yes	10	
**Subtotal score**		**/230**	

Component 2: Environmental control measures

2.1. Is there any natural and or mechanical ventilation in place, especially in waiting areas, examination room, sputum collection room, laboratory, and patient wards?	☐ No	0	
	☐ Yes	5	
2.2. Is there any outdoor waiting space or area for suspected or TB patients provided at the health facility?	☐ No	0	
	☐ Yes	5	
2.3. Are there fans provided in the TB wards?	☐ No or don't know	0	

	☐ Yes	5	
2.4. Is the TB ward doors and windows opened every day to improve air circulation?	☐ No	0	
	☐ Yes, sometimes	2.5	
	☐ Yes, always	7.5	
**Subtotal score**		**/25**	

Component 3: Personal Respiratory control measures

3.1. Are N92 and N95 respirators available for staff at the health centre? Choose one answer	☐ No or don't know	0	
	☐ Sometimes, available but inadequate numbers	2.5	
	☐ Yes, always available in adequate numbers	7.5	
3.2. Is personal protective equipment such as N92 & 95 respirators, medical face masks, gowns, boots and safety google available at all times and in sufficient quantity for all uses for all health care workers? Choose one answer	☐ No or don't know	0	
	☐ Yes, but not continuously available in sufficient quantities	2.5	
	☐ Yes, continuously available in sufficient quantities	7.5	
3.3 Do health workers re-used the personal protective equipment such as medical masks and respirators or comply with national department of health and international standards?	No or don't know	0	
	Yes	5	
3.3 Are personal protective equipment like respirators, medical masks, gowns, boots, and safety googles used	No	0	

appropriately by the health workers all the time?			
	Yes, but not used appropriately	2.5	
	Yes, appropriately used all the time	7.5	
3.4 Do patients and visitors use personal protective equipment such as medical masks while at the rural hospital?	No	0	
	Yes	10	
3.3. Does the outpatient, TB ward, x-ray and pathology use personal respirators or protection?	☐ No or don't know	0	
	☐ Yes, but not continuously available in sufficient quantities	2.5	
	☐ Yes, continuously available in sufficient quantities	7.5	
3.4. Is fit testing conducted regularly?	☐ No or don't know	0	
	☐ Yes	10	
3.5 Are healthcare workers trained to fit check for respirators with each use?	No or don't k ow	0	
	Yes	10	
3.6. Is there a cough officer appointed to provide cough hygiene education to patients and guardians at the health facility?	☐ No	0	
	☐Yes	10	
**Subtotal score**			**/50**

Interpretation: A three-step process

4. **Add up your points**

	**Score**
Section (Core component)	Subtotals
1. Managerial/Administrative control measures	230
2. Environmental control measures	25
3. Personal respiratory control measures	50
**Final total score**	**/305**

5. **Determine the assigned "TBIC level" in your facility using the total score from Step 1**

Total score (range)	TBIPC level
0–75	Inadequate
76-150	Basic
151-230	Intermediate
231-305	Advanced

6. **Review the framework results and develop an action plan**

Review the areas identified by this evaluation as requiring improvement in your facility and develop an action plan to address them. To undertake this task, consult the WHO *Interim practical manual* supporting implementation of the WHO Guidelines on Core Components of Infection Prevention and Control Programmes which will provide you with guidance, templates, tips, and examples from around the world as well as with a list of relevant IPC improvement tools. Keep a copy of this assessment to compare with repeated uses in the future.

Thank you for your participation. End of survey
