# Supplementary material for: Tuberculosis infection prevention and control in rural Papua New Guinea: an evaluation using the infection prevention and control assessment framework
Source: Antimicrob Resist Infect Control. 2023 Apr 12;12:31. doi: 10.1186/s13756-023-01237-9 (PMC10092912; doi:10.1186/s13756-023-01237-9)
Supplement: Supplementary file 2 — Supplementary information 2: TB infection control practice observation checklist. [file 13756_2023_1237_MOESM2_ESM.pdf]

Observation checklist

Section 1: Health facility demographics

1.1 Name of the health facility	
1.2 Address	
1.3 District/LLG/Province	
1.4 Region – Momase/Highlands	
1.5 Name of responsible person for infection control in this facility	
1.6 Participant’s name	
1.7 Age	
1.8 Sex	
1.9 Position	
1.10 Contact details	
1.11 Years of work at health facility	
1.12 Educational qualification (MO,HEO,NO,CHW,Others)	
1.13 Facility ownership – Church/Government/Private	

Section 2: Observation of TBIC measures in this health facility

Adopted from 2019 WHO checklist for periodic evaluation of TBIC in healthcare facilities

**Component 1: Administrative control measures**	**Day 1**			**Day 2**			**Day 3**		
	Consistent/ Always	Not consistent/ Occasionally	Not at all	Consistent/Always	Not consistent	Not at all	Consistent/Always	Not consistent	Not at all
1. Which of the following recommended controls are practiced?									
Triage									
Separation									
Cough etiquette/screening									
Prompt service for coughers									
2. Is there a systematic screening for patients for cough?									
3. Are patients with cough separated early from other patients?									
4. Is there IEC regarding cough etiquette on site?									
5. Are masks & tissues provided for coughing patients?									
**Component 2: Environmental control measures**	Consistent/ Always	Not consistent/ Occasionally	Not at all	Consistent/Always	Not consistent	Not at all	Consistent/Always	Not consistent	Not at all
6. Maintain natural and mechanical ventilation system									
Waiting room (indoor)									
Examination room									
Sputum collection room									
Patient ward									
Isolation room									
7. Is there any outdoor waiting area for suspected or TB patients provided?									
8. Are there fans provided in the TB ward?									
9. Is the TB ward doors and windows opened every day to monitor air circulation?									
**Component 3: Personal respiratory protection**	Consistent/ Always	Not consistent/ Occasionally	Not at all	Consistent/Always	Not consistent	Not at all	Consistent/Always	Not consistent	Not at all
10. Are respirators available for all staff at the health center?									

11. Which respirator model/type is used in which department?									
12. Does the outpatient, TB, ward, x-ray and pathology use respirators?									
13. Is there a cough officer appointed to provide cough hygiene and education at the heath facility?									

End of observation!!
